# Relative Rates of Gluten Digestion by Nine Commercial Dietary Digestive Supplements

**DOI:** 10.3389/fnut.2021.784850

**Published:** 2021-12-07

**Authors:** Gregory John Tanner

**Affiliations:** School of Biosciences, University of Melbourne, Melbourne, VIC, Australia

**Keywords:** gluten digestion, proline endopeptidase, AnPEP, caricain, DPP-IV, celiac disease

## Abstract

Endopeptidases containing supplements may digest gluten and reduce the impact on celiac and gluten-sensitive subjects who inadvertently consume gluten. We investigated the relative rate of disappearance of coeliac relevant epitopes in extracts of nine commercial supplements, using two competitive enzyme-linked immunosorbent assays (ELISAs)—Ridascreen (detects QQPFP, QQQFP, LQPFP, and QLPFP) and Gluten-Tec (detects Glia-α20 and PFRPQQPYPQ). All epitopes are destroyed by cleavage after P and Q amino acids. Rates at pH 3.5 and pH 7.0 were measured. These experiments were designed to measure relative rates of epitope digestion not to mimic *in vivo* digestion. The supplements were: 1 GluteGuard, 2 GlutenBlock, 3 GliadinX, 4 GlutnGo, 5 GlutenRescue, 6 Eat E-Z Gluten+, 7 Glutenease, 8 Glutezyme, and 9 Gluten Digest. The mean initial rate and half-lives of epitope digestion were deduced and extrapolated to rates at the recommended dose of one supplement in a fasting stomach volume. At pH 7, supplement 1 was the fastest acting of the supplements, with Ridascreen ELISA, more than twice as fast as the next fastest supplements, 5, 6, 7, and 8. Supplements 2, 3, and 4 showed little activity at pH 7.0. Supplement 1 was also the fastest acting at pH 7 with Gluten-Tec ELISA, more than three times the rate for supplements 2 and 3, with supplements 4–9 showing minimal activity. At pH 3.5, supplement 1 acted more than five times as fast as the next fastest supplements, 2 and 3, when measured by Ridascreen, but supplements 2 and 3 were over two times faster than supplement 1 when measured by Gluten-Tec. Supplements 4–9 demonstrated minimal activity at pH 3.5 with either ELISA. Supplement 1 most rapidly digested the key immuno-reactive gluten epitopes identified by the R5 antibody in the Codex-approved competitive Ridascreen ELISA method and associated with the pathology of celiac disease.

## Introduction

Celiac disease (CD) occurs when the extensive immune system associated with the human intestine reacts to dietary gluten (1, 2). The condition occurs when particular nine amino-acid epitopes are presented to the intestinal lamina propria. These epitopes are well-defined (3–5). This process is activated by the introduction of negatively charged residues into gluten peptides, following the action of human tissue transglutaminase which converts glutamine (Q) residues into negatively charged glutamic acid (E) residues (1, 2).

Gluten consumption by celiac promotes an extensive cascade of immune reactions that ultimately results in the destruction of the intestinal villi. CD leads to increased rates of intestinal malignancy and a raft of adverse health outcomes (6, 7). Retrospective analysis of stored blood samples for immunological markers of CD shows that ~1% of most populations suffer from CD (8). Up to half of the celiacs remains undiagnosed (9).

In addition to celiacs, ~10% of the population report avoiding gluten, with symptoms similar to irritable bowel syndrome (10). These subjects may suffer from non-celiac gluten intolerance (11, 12). A smaller subset of the population, ~0.5%, suffers from a wheat allergy, a rapid onset, life-threatening IgE mediated reaction to gluten (13).

Sufferers of all three gluten-related conditions must maintain a lifelong avoidance of gluten (14). However, a gluten-free (GF) diet has many nutritional shortcomings (15), for example, preliminary studies indicate that prolonged consumption of a GF diet is associated with adverse changes in the microbiome (16).

Gluten resists complete digestion by the human intestinal digestive endo-proteases, pepsin, trypsin, chymotrypsin, and carboxypeptidase due to the high content of proline residues. Undegraded immuno-reactive peptides, such as the 33-mer α2-gliadin peptide (Glia 57-89; LQLQPFPQPQLPYPQPQLPYPQPQLPYPQPQPF) (2), and the 26-mer γ5-gliadin peptide (Glia 26-51; FLQPQQPFPQQPQQPYPQQPQQPFPQ) (17), interact with the lamina propria, initiating the celiac cascade.

Two-thirds of coeliacs on a well-controlled, GF diet are inadvertently exposed to dietary gluten contamination (18). A potential solution to inadvertent gluten consumption exists by supplying dietary proteases, which are active at physiological pH of 3.5 (stomach) or pH 7 (intestine), and resistant to pepsin and trypsin. These supplements must be capable of rapidly hydrolyzing immuno-reactive peptides, in particular, those containing X-proline bonds before they reach the intestine. The supplements must also cleave the immuno-reactive peptides into small enough fragments that remain inactive in the intestine (19).

A survey of the enzyme activity in five commercially available digestive enzyme supplements by enzyme assays, gluten epitope degradation monitoring by R5 the enzyme-linked immunosorbent assay (ELISA) and mass spectrometric (MS) analysis of the degradation products, and toxicity remaining monitored by T-cell proliferation assays was reported (20). This study found that most supplements were largely ineffective. Other surveys also predicted that many supplements were ineffective (21, 22).

We evaluated enzyme supplements that were commercially available at the time of this work. Several additional supplements in development were not evaluated. These include a recombinant glutamine-specific endoprotease [EP-B2 from barley (23)], a *Sphingomonas capsulata* prolyl endopeptidase termed ALV003, now Latiglutenase (ImmunogenX, Newport Beach, CA, USA) (ImmunogenX, Newport Beach, CA, USA) (24, 25), a recombinant *Alicyclobacillus sendaiensis* serine endopeptidase [Kuma30 now Tak-062 (Takeda Pharmaceutical Company Limited, Japan, Feb 2020) (26), and an endopeptidase 40 (E40) (27).

Some digestive supplements contain dipeptidyl peptidase IV (DPP-IV), an X-Pro amino-exopeptidase from *Aspergillus oryza*e, inactive at stomach pH 3.5, but optimally active at intestinal pH 7.0 (28). DPP-IV was resistant to pepsin and only releases proline-containing dipeptides from the N-terminus (29). DPP-IV, in combination with *Aspergillus niger* aspergillopepsin, degrades small amounts of gluten *in vitro* (29). MS-based techniques showed small amounts of immuno-reactive fragments remained after digestion (20).

Other supplements employ food-grade *Aspergillus niger* prolyl endopeptidase (AnPEP, also Tolerase G, supplied by DSM, Kaiseraugst, Switzerland). AnPEP is resistant to pepsin, active at stomach pH 3.5, inactive at intestinal pH 7.0, and effective in digesting gluten *in vitro*. AnPEP cleaves gluten peptides specifically on the C-terminus of proline residues (30–32). AnPEP digests the 33-mer α-gliadin (33). Gluten was degraded by AnPEP before entering the duodenum in complex human meal situations (34, 35). Human trials have shown that AnPEP is tolerated but AnPEP consumption did not demonstrate a protective effect on symptoms (36).

Caricain (EC 3.4.22.30) from *papaya* latex (37) is a component of the most active supplement, supplement 1 (GluteGuard). The enzyme was first described by Schack (38). Caricain cleaves the C-terminus of proline residues (39). Caricain cleaves purified gliadin (40) and gliadin in whole wheat flour (41) and detoxified gliadin extracts (42). Caricain appears suitable as a basis for enzyme treatment of CD (43). Clinical studies have shown that caricain supplements reduce gluten-induced symptoms. There are two key randomized, double-blind, placebo-controlled studies, firstly in patients with dermatitis herpetiformis (44) and secondly in celiacs (45). The first study measured the skin lesion size. Patients were challenged with 6 g of gluten daily for 14 days. Ten subjects received a placebo and 10 subjects received GluteGuard before each gluten challenge. Skin lesion size was significantly reduced by the treatment. The second study involved celiac patients in remission. Six patients were assigned to the placebo group, 14 to the caricain group who received GluteGuard. All subjects received 1 g of gluten daily for 45 days. Four of the placebo subjects abandoned the study due to severe symptoms. Treatment significantly protected against gluten-induced symptoms.

Here we examined the relative rates of gliadin consumption measured by Ridascreen R5 and Gluten-Tec competitive ELISA by extracts of nine commercially available digestive supplements at pH 3.5 and pH 7.

## Materials and Methods

### Commercial Gluten-Digesting Enzyme Supplements

Nine commercially available gluten-digesting enzyme supplements were obtained and stored, dry at 4°C. The contents of each supplement are described in Table 1, the protein concentration of enzyme extracts is described in Supplementary Table 1.

**Table 1:** Commercially available enzyme supplements evaluated.

**No**.	**Supplement**	**Active Ingredient**	**Manufacturer**
1	GluteGuard	300 mg oleoresin (Caricain)	Glutagen Pty Ltd. Melbourne, Australia.
2	GlutenBlock	400 mg Tolerase^®^G Neutral protease 6000 PC/g Prolyl endopeptidase (*Aspergillus niger*) 232,000 PPI	Pharmacist Formulas, Alpharetta, USA
3	GliadinX	335 mg Prolyl endopeptidase (*Aspergillus niger*)	AVI Research, Chicago, USA
4	GlutnGo	100 mg Tolerase^®^G Prolyl endopeptidase (*Aspergillus niger*)	Brickerlabs, Chandler, USA
5	Gluten Rescue	350 mg Glutalytic (Plant protease mix) Aspergillopepsin 500 SAPU Protease DP-PIV 125 DP-PIV Protease 75,000 HUT	Doctors Best Inc. San Clemente, USA
6	Eat E-Z Gluten+	DP-PIV 1,100 DPPU Protease (I, II, III, IV, V) 155,150 HUT	Dynamic Enzymes, Anaheim Hills, USA
7	Glutenease	DP-PIV 1,000 DPPU Protease Thera-blend^TM^ 95,000 HUT	Enzymedica, Venice, USA
8	Glutezyme	Acid fungal protease (*Aspergillus niger*) 500 SAPU Bacterial protease (*Bacillus subtilis*) 50,000 PC Bromelain (*Ananas comosus*) 100,000 PC *BioCore DPP IV*: Protease A. oryzae 167 DPP_IV :Protease (A. oryzae) 10,000 HUT :Protease (A. mellieus) 2.8 AP	Progressive Laboratories Inc. Irving, USA
9	Gluten Digest	BioCore DPP-IV: Protease (*Aspergillus oryzae*) 30,000 HUT :Protease (*Aspergillus oryzae*) 500 DPP-IV : Protease (*Aspergillus mellieus*) 8.5 AP	Now Foods, Bloomingdale, USA

### Urea-Sodium Dodecyl Sulfate–Polyacrylamide Gel Electrophoresis (SDS-PAGE) and Western Blots

Proteins were analyzed by Urea-SDS-PAGE and either stained with 0.06% (w/v) colloidal Coomassie Blue or electro-blotted to nitrocellulose membranes (iBlot2, Novex) developed with anti-gliadin-HRP 1/1,000× (Sigma), detected by chemiluminescence (Amersham), and calibrated against pre-stained proteins (Invitrogen) as described (46).

### Isolation of Gliadin and Glutenin Subunits From Wheat cv Baxter

**Figure 1:**
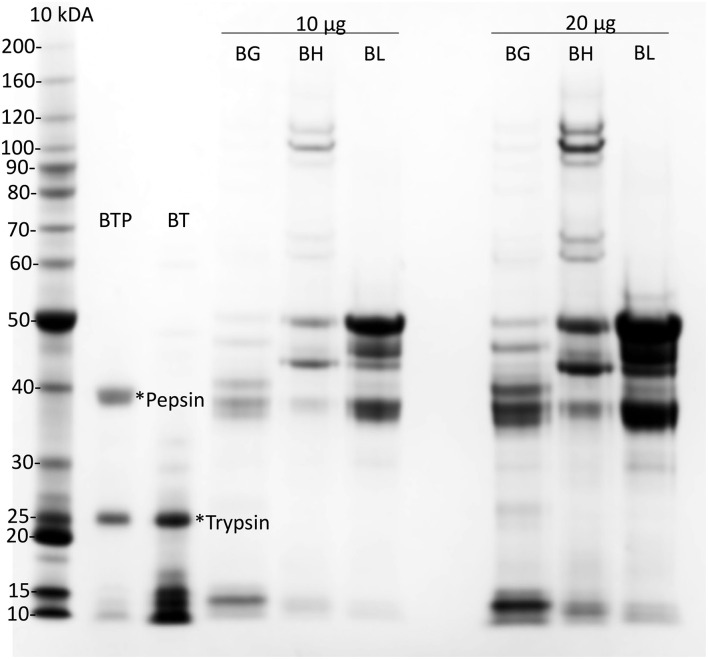
Preparation of cv Baxter gliadin. Preparations of cv Baxter gliadin (*BG*), HMWGS (*BH*), and LMWGS (*BL*) containing 10 μg (*10* μ*g*) and 20 μg (*20* μ*g*) of protein were resolved by Urea-SDS-PAGE. Gliadin was digested with trypsin (*Trypsin) and then pepsin (*Pepsin) and protein resolved by Urea-SDS-PAGE from the trypsin (*BT*) and trypsin/pepsin (*BTP*) digests of gliadin. Proteins were stained with Coomassie Blue and calibrated against standard proteins (Invitrogen, 10 kDa ladder). SDS-PAGE, sodium dodecyl sulfate–polyacrylamide gel electrophoresis.

Gliadin was purified from a single wheat cultivar, cv Baxter, from 10 g white flour, as described (47). Freeze-dried cv Baxter gliadin was dissolved at 50 mg/ml in 8 mol/L urea, 1% (w/v) dithiothreitol (DTT), and 20 mmol/L triethylamine-HCl pH 6 (Urea-DTT-TEA). High molecular weight and low molecular weight glutenin subunits (HMWGS and LMWGS, respectively), were isolated from the pellet remaining after gliadin extraction, as described (47). These subunits were prepared to assist in the evaluation of the purity of the isolated gliadin (Figure 1).

### Preparation of Trypsin/Pepsin-Treated cv Baxter Gliadin (TP-Gliadin)

Purified gliadin (cv Baxter) 370 mg, was dissolved in 7.4 ml of Urea-DTT-TEA, diluted ~25 × to 200 ml with MilliQ water (to reduce the urea concentration below 320 mmol/L, where trypsin is active) (46) and 25 mg of trypsin (Sigma TPCK treated bovine pancreas in 1 mmol/L HCl) added to give trypsin: gliadin ratio of 1: 20. The pH was adjusted to 7, and the gliadin was digested with trypsin for 2 h at RT during which time the initially cloudy suspension cleared. The pH was adjusted to 3.0 with 1 mol/L HCl, 50 mg of pepsin (Sigma, porcine gastric mucosa in 1 mmol/L HCl, 50% v/v glycerol) added, pH readjusted to 3.0, and the trypsin-treated gliadin digested over-night at RT. The clear digest was freeze-dried and re-dissolved in the original volume of MilliQ water to re-establish a protein concentration equivalent to 50 mg/ml (TP-gliadin). The TP-gliadin was examined by SDS-PAGE and high-performance liquid chromatography (HPLC) (Figure 1 and Supplementary Figure 3). This pre-digestion process was the reverse of that encountered physiologically where pepsin digestion at pH 3 would occur first. This was done deliberately so that residual peptides would be more soluble at the more acid pH due to residual buffer when reconstituted in MilliQ water as above. The reverse treatment, i.e., pre-digestion with pepsin and then trypsin (PT-gliadin), produced a less stable peptide solution which tended to precipitate after storage at 4°C or thawing after freezing, requiring a fresh PT-gliadin preparation each day which was impractical. The changed presentation of peptides each day would make a comparison of kinetic rates on different days impossible. TP-gliadin solution was stable, and the solution could be aliquoted, frozen, and thawed when required. This presented exactly the same concentration and composition of peptides each day, a basic requirement for relative kinetic analysis of digestions in different experiments. Although the peptide patterns of the two alternate gliadin preparations differ on HPLC-MS/MS (Colgrave pers. commun.), there was no significant difference in the rate of digestion by supplement 1, when digestion of the two alternate gliadin hydrolysates was compared with Ridascreen ELISA. This is interpreted as follows: HPLC-MS identifies the composition of individual peptides, so peptides that differ by one or two residues between the two treatments can be resolved and identified. On the other hand, ELISA is an averaging technique—so not one epitope is measured but the response is averaged over many epitopes on many peptides, minimizing the differences in rates of digestion of the two gliadin preparations measured by ELISA.

### Digestion of Trypsin/Pepsin Treated cv Baxter Gliadin by Supplements

Relative kinetic constants of epitope digestion were calculated for the nine supplements. This enabled the comparison of the relative rates of gliadin digestion by enzyme supplements with the recommended enzyme dose of one supplement in a fasting stomach volume of 100 ml (48). Therefore, a working (1×) concentration of enzyme consisted of one pill in 100 ml. The final gliadin concentration in digests was the equivalent of 0.25 g/100 ml of stomach volume.

The final enzyme concentration was reduced in reactions, so that the kinetic parameters of gliadin epitope hydrolysis (initial velocity and half-life) could be accurately calculated. These kinetic parameters were then extrapolated to a final enzyme concentration of 1× as below so that the relative initial velocities could be compared. The final pH was either pH 3.5 or 7.0 to allow examination of the effect of pH on the rate of gliadin consumption. These pH values are similar to those used to simulate stomach or intestinal gluten digestion (49) however, the experiments reported here are not intended to mimic *in vivo* digestion conditions, rather allow calculation of relative rates of physiologically relevant epitope consumption.

Duplicate tablets of digestive supplement 1 were ground to a powder in a mortar and pestle and prepared as 11.1× concentrated solutions by pouring the powders into 9 ml of 50 mmol/L Na acetate, 50 mmol/L NaCl, adjusted to pH 2.5 (MM3.5 buffer), designed to yield a final pH 3.5 in the enzyme reaction. Duplicate extracts of supplements 2–9 (powdered in capsule form) were made by pouring the powders into 9 ml of MM3.5. Contents were mixed by vortexing 1 min, regularly inverting the tube every 10 s for 30 min at RT, and clarified by centrifugation at 2,000 *g*/ 5 min. It was essential to use MM3.5 buffer to extract active enzymes from AnPEP-containing pills. No activity was observed if AnPEP supplements were extracted at pH 7 (Supplementary Figure 1), at either RT or 4°C. The addition of 500 mmol/L NaCl did not improve the extraction of AnPEP activity. The reason for this is unknown but may be a result of an interaction between the AnPEP enzyme and the inert binder causing inadvertent pelleting of the enzyme.

Enzyme solutions were prepared as above and diluted as required to 0.11 ×, 0.011 ×, or 0.0011 × enzyme concentrations as indicated, with an appropriate volume of phosphate-buffered saline (PBS) buffer containing 0.1% (w/v) Tween 20 (PBST). Enzyme reactions were prepared in 200 μl polypropylene ELISA wells by adding 20 μl of enzyme supplement appropriately diluted with PBST as indicated, to a final volume of 200 μl, further diluting the supplement concentration by 0.1 ×. The reaction contained 168 μl of either MM3.5 or MM7 buffer (50 mmol/L Na-phosphate, 50 mmol/L NaCl adjusted to pH 7.0 and designed to yield a final pH 7.0 in the enzyme reaction) and 12 μl of TP-gliadin (41.8 mg/mL in Urea-DTT-TEA). Enzyme alone (EA) control solutions contained 20 μl of appropriately diluted supplement as above, diluted to a final volume of 200 μl by addition of 168 μl of either MM3.5 or MM7, and 12 μl of Urea-DTT-TEA in place of cv Baxter gliadin. A zero-time control consisted of 168 μl of either MM3.5 or MM7, 12 μl TP-gliadin (41.8 mg/ml in Urea-DTT-TEA), and 20 μl of PBST in place of an enzyme. Reactions were equilibrated at 30°C with gentle agitation. The proteolysis was started by the addition of enzyme unless noted, and 20 μl aliquots were taken at the indicated time from 10 to 60 min, diluted by either 1/1,000× (Gluten-Tec Elisa) or 1/4,000 (Ridascreen Elisa) with PBST. PBST was used in place of the Gluten-Tec sample diluent, which produced a gel upon heating which prevented accurate pipetting. Diluted reaction aliquots (100 μl) were transferred to PCR tubes, heated at 95°C for 20 min to eliminate any residual proteolytic activity. The solutions were cooled, centrifuged briefly to collect droplets from the lid, and aliquots (40 μl) were taken for ELISA as below.

Digestions for western blot analysis were as above but contained either 0.5 mg of Baxter gliadin or TP-gliadin but at 1.1× enzyme concentration (i.e., 10× higher than for kinetic experiments) at both pH 3.5 and pH 7.0.

### Kinetics of Digestion of Coeliac Epitopes Estimated With Gluten-Tec Competitive ELISA

For the Gluten-Tec competitive ELISA (EuroProxima B.V., Arnhem, The Netherlands), dilute, heat-treated reaction aliquots (40 μl) were further diluted with 10 μl PBST and added to ELISA wells with 50 μl 1/100× dilute antibody-horseradish peroxidase conjugate and left at 4°C for 3 h to equilibrate with gentle agitation. ELISA wells were rinsed 5× with manufacturer wash buffer and tapped dry. Manufacturer substrate (100 μl) was added and left to react at 30°C for 15 min and stopped with 100 μl stop reagent. The A450 of wells was read on a Perkin Elmer plate reader and calibrated against 0–210 ng dilutions of cv Baxter gliadin (Supplementary Figure 2C).

### Kinetics of Digestion of Coeliac Epitopes Estimated With Ridascreen Competitive ELISA

The Ridascreen competitive ELISA was performed as per the manufacturer's instructions (R-Biopharm AG, Darmstadt, Germany). Dilute, heat-treated reaction aliquots (40 μl) were diluted with 10 μl PBST and added to Ridascreen Competitive ELISA wells with 50 μl of 1/10× dilute antibody-horseradish peroxidase conjugate and left at 30°C for 30 min to equilibrate with gentle agitation. ELISA wells were rinsed 5× with manufacturer wash buffer and tapped dry. Manufacturers substrate (100 μl) was added and left to react at 30°C for 10 min and stopped with 100 μl of stop reagent. The A450 was read with a Perkin Elmer plate-reader and calibrated against 0–30 ng dilutions of cv Baxter gliadin (Supplementary Figure 2A).

### ELISA Controls

Dilute and heat-treated solutions (10 min at 95°C) still damaged the ELISA antibodies, due to residual proteolytic activity from the supplements. This was demonstrated by decreased signal levels (Supplementary Figures 2B,D). No damage was evident when diluted reactions were heated for 20 min at 95°C.

### Calculations

Duplicate absorbances from the plate reader were transformed into amounts of Baxter gliadin using the appropriate standard curve and biological duplicates plotted vs. time using GraphPad Prism (version 8.4.2 for Windows, GraphPad Software, San Diego, CA, USA, www.graphpad.com). The decreasing gliadin amounts remaining over time were fitted to first-order non-linear decay models and initial rate (±SE) and half-life (±SE) calculated. In general, the data well-fitted the curves with *R*^2^ values >0.9. However, where rates of digestion were low and the data were noisy, fitting data to non-linear regressions did not return reasonable estimates of initial rate or half-life, and the data were therefore fitted to linear regressions which returned more reasonable estimates of initial rate and half-life. With linear regressions, only the SE of the initial rate could be calculated by the software.

More rapid gliadin hydrolysis was indicated by a higher initial rate and shorter half-life. The initial rate and half-life of gliadin digestion were normalized to enzyme concentrations of 1× by dividing the observed initial rate and SE by the dilution and multiplying the observed half-life and SE by the dilution. The significance of differences in normalized mean initial rate and half-life were examined by the one-way ANOVA with Tukey's multiple comparison test at the indicated significance level.

### Protein Concentrations of Enzyme Solutions

Protein concentrations of the nine enzyme supplements were determined by the method of Bradford, calibrated against gamma-globulin (50). The protein concentration varied from 0.23 mg/ml (Supplement 4) to about 1 mg/ml (Supplements 1, 6, 7, 8, and 9, Supplementary Table 1); however, the dose of digestive supplements was related to a dose of one pill dissolved in 100 ml.

## Results

### Preparation and Digestion of Gliadin

Gliadin was isolated from a single wheat cultivar cv Baxter. Analysis by Urea-SDS-PAGE showed that the gliadin was uncontaminated with HMWTGS and LMWTGS (Figure 1, *BG*). Urea-SDS-PAGE of trypsin-treated gliadin showed in addition to a trypsin band at 25 kDa (Figure 1, lane *BT*, *trypsin) a number of small peptides that ran at <17 kDa (Figure 1, *lane BT*). Urea-SDS-PAGE of TP-gliadin produced in addition to trypsin and pepsin bands (Figure 1, lane *BTP*, *trypsin and *pepsin, at 25, and 39 kDa, respectively), smaller peptides that ran ahead of the electrophoretic front <10 kDa. HPLC showed that the TP-gliadin consisted of over 40 small peptides (Supplementary Figure 3) and confirmed digestion.

**Figure 2:**
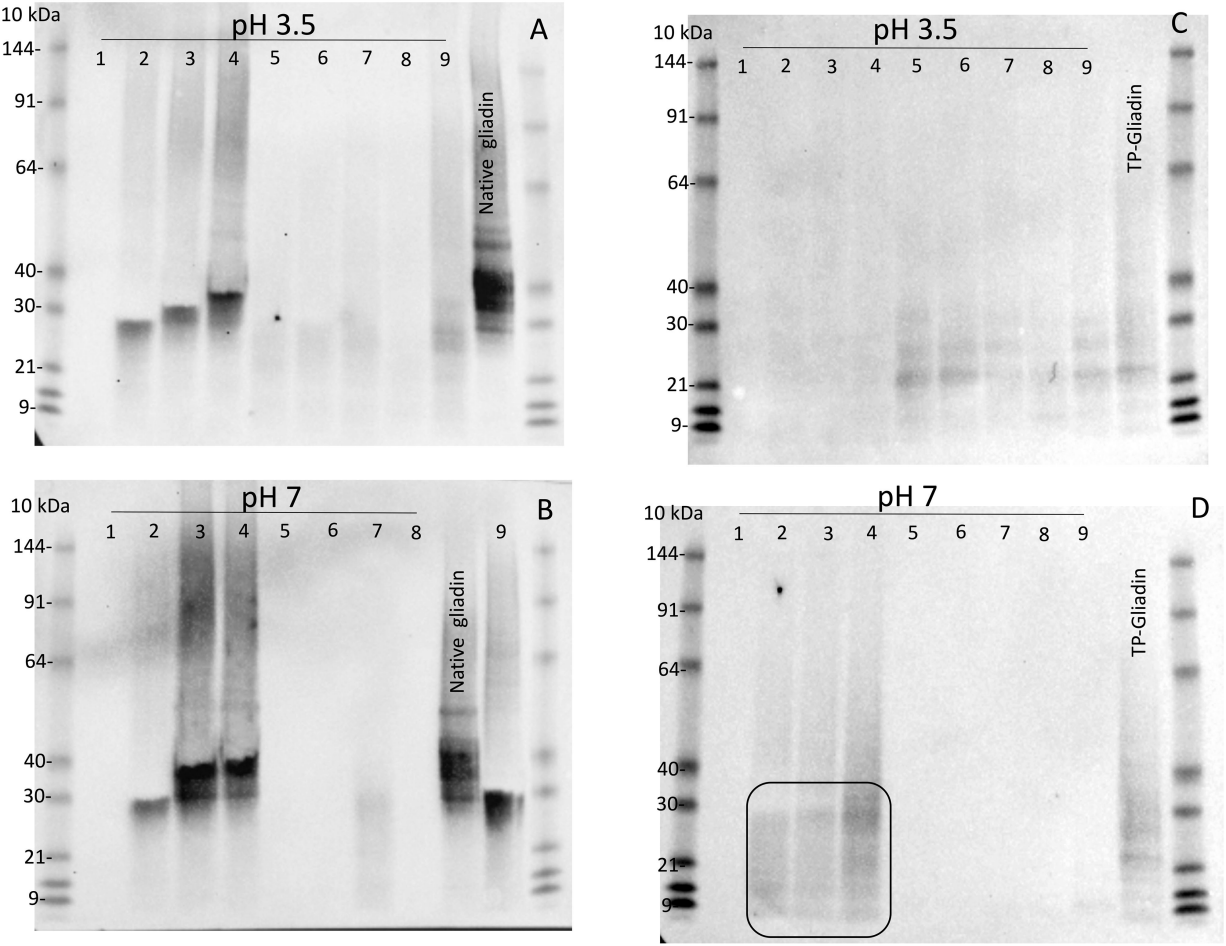
The activity of supplements 1–9, with native gliadin and TP-gliadin by western blot. Native Sigma gliadin **(A,B)** or TP-gliadin **(C,D)** was digested at either pH 3.5 **(A,C)** or pH 7 **(B,D)** with a final enzyme concentration of 1.1× from supplements 1–9 as indicated for 60 min at 30°C and compared to native gliadin (Sigma, *Native gliadin*) or cv Baxter TP-gliadin (*TP-gliadin*) by Urea-SDS-PAGE. Proteins were blotted to nitrocellulose, developed with anti-gliadin-HRP, detected by chemiluminescence, and calibrated against pre-stained standard proteins (*10 kDa*). A volume equivalent to 2 μg of original protein was loaded on each lane. Digestion is shown by the absence of western bands. SDS-PAGE, sodium dodecyl sulfate–polyacrylamide gel electrophoresis.

Digestion of both native gliadin and digested TP-gliadin was firstly examined by western blotting of Urea-SDS-PAGE gels of all extracts, but at 1.1× enzyme concentration (i.e., 10× higher than for kinetic experiments) at both pH 3.5 and pH 7 (Figure 2). Lanes were loaded with equal protein loads equivalent to 2 μg of protein. Enzyme activity was demonstrated by the absence of western bands for native or TP-gliadin. Native gliadin runs as a series of dominant bands at 30–40 kDa. The TP-gliadin runs largely at the electrophoretic front with a small amount of partially degraded peptides in the range 20–30 kDa (e.g., Figure 2D, *ellipse*). Supplement 1 was active under all conditions, removing both native and TP-gliadin fragments. At this high enzyme concentration, the DPP-IV supplements 5, 6, 7, and 8 were also active with native gliadin at both pH (Figures 2A,B). Supplements 2, 3, and 4 had no activity with native gliadin (Figures 2A,B) as expected for AnPEP enzymes. Supplement 9 was active with native gliadin but only at pH 3.5 (Figure 2A) and appeared active with TP-gliadin but only at pH 7. The AnPEP supplements 2, 3, and 4 were active with TP-gliadin at pH 3.5 (Figure 2C) but had no activity at pH 7 (Figure 2D) as expected. The DPP-IV supplements 5, 6, 7, and 8 were only active with TP-gliadin at pH 7 (Figure 2D) and had little activity at pH 3.5 (Figure 2C).

### Stability of Enzyme Preparations

Residual proteolytic activity in diluted samples had to be inactivated before ELISA assay to avoid damage to the ELISA antibodies. Proteolytic activity was remarkably stable and resisted most methods commonly used to destroy proteolytic activity. Adjustment of supplements 1, 2, 6, 7, and 8 to pH 12.3 (final 1 mol/L NaOH) for 20 min did not inhibit subsequent gliadin proteolysis. Dilution to final concentrations of 2% (w/v) SDS, 1% (w/v) DTT, and heating at pH 7 at 95°C/5 min did not inhibit the subsequent hydrolysis of gliadin by supplement 1 but did inhibit the activity of supplement 2. Incubation of supplement 1 in final concentrations of 4 mol/L urea, 1% (w/v) SDS for 30 min at RT did not inhibit hydrolysis of gliadin. Incubation in 80% (v/v) acetone at 70°C for 30 min did not inhibit gliadin proteolysis in supplements 1, 5, 6, 7, or 8. Supplements 1, 2, 5, and 6 heated at 95°C/10 min still had slight activity, whereas all other supplements were inhibited by this treatment. It was necessary to heat the final dilutions at 95°C/20 min to remove all remaining proteolytic activity from all supplements (Supplementary Figure 2). Supplement 1 was stable to the action of both trypsin and pepsin (Supplementary Figure 4).

### Kinetics of Digestion of Coeliac Epitopes Estimated by Gluten-Tec ELISA

**Figure 3:**
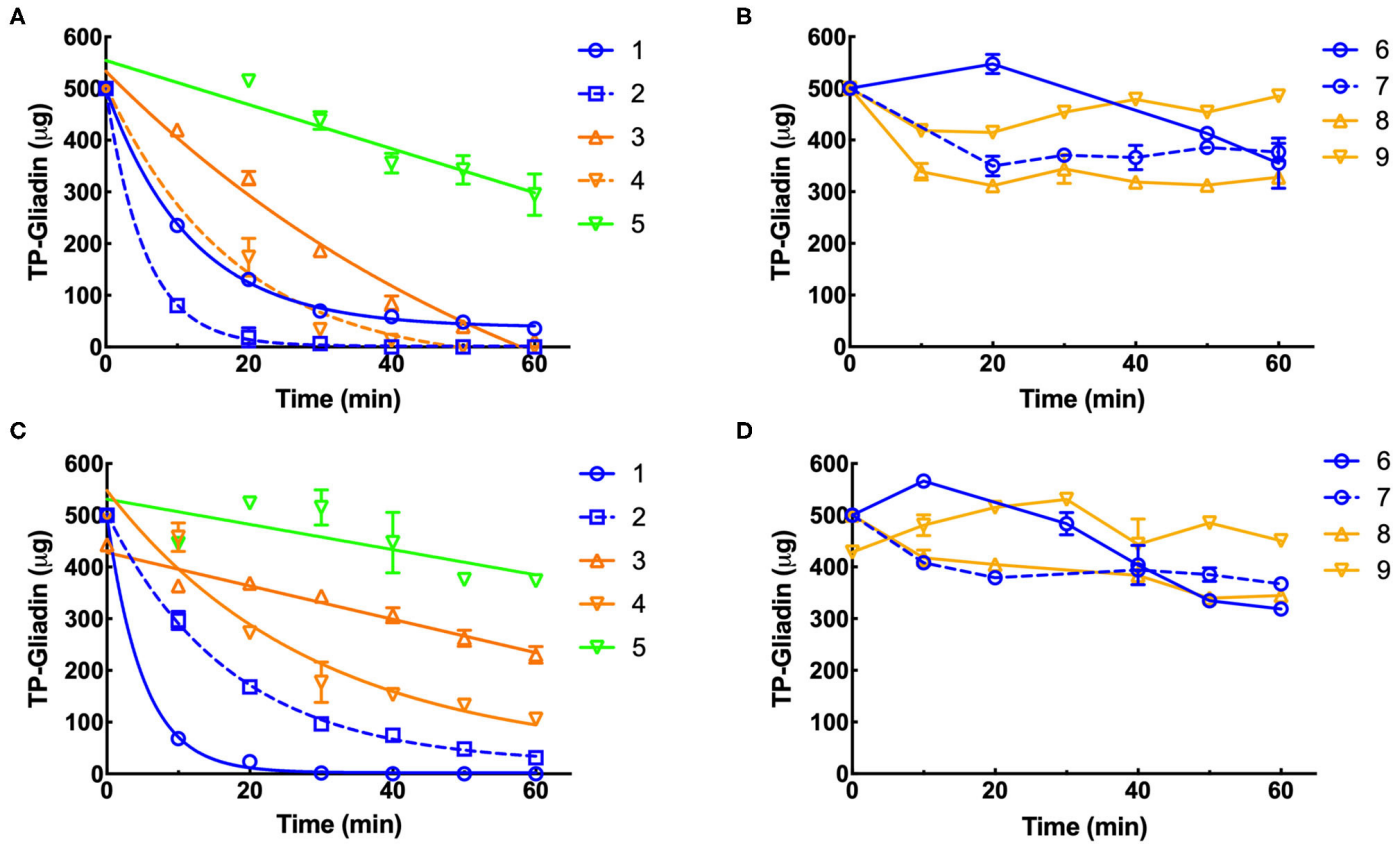
Kinetics of digestion of coeliac epitopes estimated by Gluten-Tec ELISA. Duplicate extracts of supplements 1–9 were diluted to a final enzyme concentration of 0.11×, except supplement 3 (0.011×). The final enzyme concentration was reduced in reactions so that the kinetic parameters of the reaction (initial velocity and half-life) could be accurately calculated. Dilute enzymes were added to reaction mixtures and gluten digestion commenced. Samples were taken every 10 min as indicated, over 60 min at pH 3.5 **(A,B)** and pH 7 **(C,D)**, diluted by 1,000× with PBST, heated at 95°C for 20 min to destroy residual protease activity and remaining gliadin epitopes measured by Gluten-Tec ELISA calibrated against cv Baxter gliadin standard curve (Supplementary Figure 2C). The standard curve was used to translate raw A450 ELISA data into μg of cv Baxter gliadin remaining and the data fitted by non-linear regression: **(A)** supplements 1, 2, 3, and 4, all with *r*^2^ > 0.97 and **(C)** supplements 1, 2 all with *r*^2^ > 0.99. However, where rates of digestion were low and the data were noisy, fitting data to non-linear regressions did not return reasonable estimates of initial rate or half-life, and the data were therefore fitted to linear regressions for all other curves. For clarity, only lines joining points are shown for data analyzed by linear regression. Mean (±SE) is shown, except when an error was smaller than symbol size. PBST, phosphate-buffered saline with Tween ^®^ detergent; ELISA, the enzyme-linked immunosorbent assay.

Residual epitopes were measured in duplicate by Gluten-Tec competitive ELISA and from the lines of best fit (Figure 3), the initial rate and half-life of gliadin digestion were determined and corrected to 1× enzyme dilution at pH 3.5 (Supplementary Table 1) and pH 7.0 (Supplementary Table 2). At pH 3.5, supplements 1, 2, 3, and 4 showed the highest rates of gliadin digestion (Figure 3A) while at pH 7.0 supplements 1, 2, and 4 showed the highest rates of gliadin digestion (Figure 3C). Preparation 5 (Figure 3A) showed a modest rate of digestion and supplements 6, 7, 8, and 9 showed little activity at pH 3.5 (Figure 3B). At pH 7, supplements 3 and 5 showed modest rates of digestion (Figure 3C) but supplements 6, 7, 8, and 9 had low activity (Figure 3D).

### Kinetics of Digestion of Coeliac Epitopes Estimated by Ridascreen ELISA Analysis

**Figure 4:**
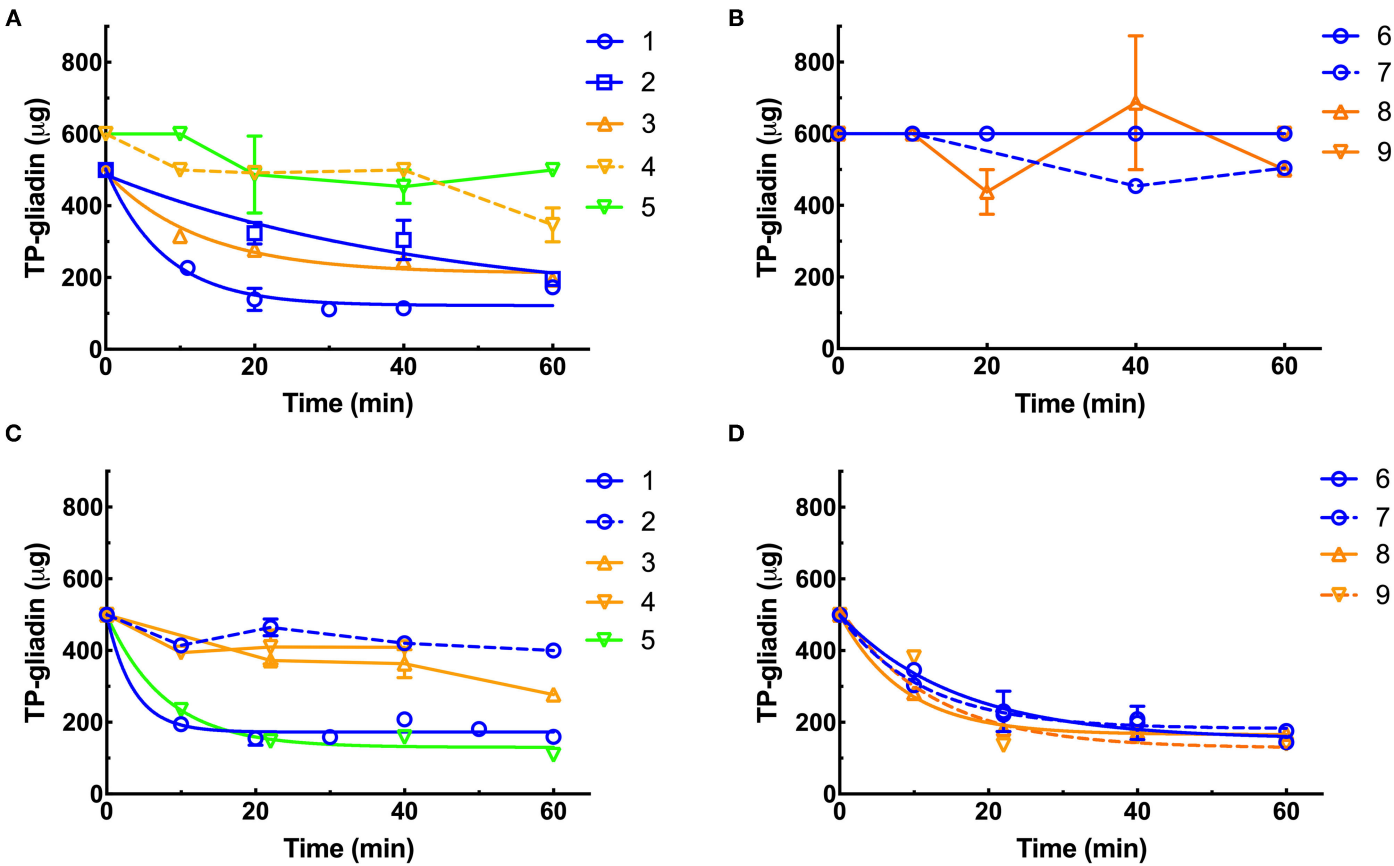
Kinetics of digestion of coeliac epitopes estimated by Ridascreen ELISA. At pH 3.5 **(A,B)**, supplements were diluted to final enzyme concentrations of 0.011×, except supplements 3, 4, and 9 (0.11×). At pH 7.0 **(C,D)**, all supplements were diluted to 0.01× except supplement 1 (0.0011×) and supplements 3, 4, and 9 (0.1×). Supplements were diluted so that the kinetic parameters of the reaction (initial velocity and half-life) could be accurately calculated. The enzymes were added to reaction mixtures and gluten digestion commenced. Samples were taken every 10 min as indicated, over 60 min at pH 3.5 **(A,B)** and pH 7 **(C,D)**, diluted by 4,000×, heated at 95°C for 20 min to destroy any protease activity and remaining gliadin epitopes measured by Ridascreen ELISA calibrated against cv Baxter gliadin standard curve (Supplementary Figure 2A). The standard curve was used to translate raw A450 ELISA data into μg of cv Baxter gliadin remaining and the data well-fitted by non-linear regressions. At pH 3.5, supplements 1, 2, and 3 all had *r*^2^ > 0.81 **(A)**. At pH 7.0 supplements 1, 5, 6, 7, 8, and 9 all had *r*^2^ > 0.88 **(C,D)**. However, where rates of digestion were low and the data noisy, fitting data to non-linear regressions did not return reasonable estimates of initial rate or half-life, and the data were therefore fitted to linear regressions for all other curves. For clarity, only lines joining points are shown for data analyzed by linear regression. Mean (±SE) is shown, except when an error was smaller than symbol size. ELISA, the enzyme-linked immunosorbent assay.

Residual epitopes were measured in duplicate by Ridascreen competitive ELISA and from the lines of best fit (Figure 4), the initial rate and half-life of gliadin digestion were determined and corrected to 1× enzyme dilution at pH 3.5 (Supplementary Table 3) and pH 7.0 (Supplementary Table 4).

At pH 3.5, supplements 1, 2, and 3 showed the highest rates of gliadin digestion (Figure 4A). Supplements 4, 5, 6, 7, 8, and 9 showed little activity at pH 3.5 (Figures 4A,B). At pH 7.0, supplements 1, 5, 6, 7, 8, and 9 showed the highest rates of gliadin digestion (Figures 4C,D). At pH 7.0, supplements 2, 3, and 4 showed low rates of digestion (Figure 4C) with Ridascreen ELISA.

### Statistical Analysis of the Kinetics of Epitope Digestion Analyzed by ELISA

**Figure 5:**
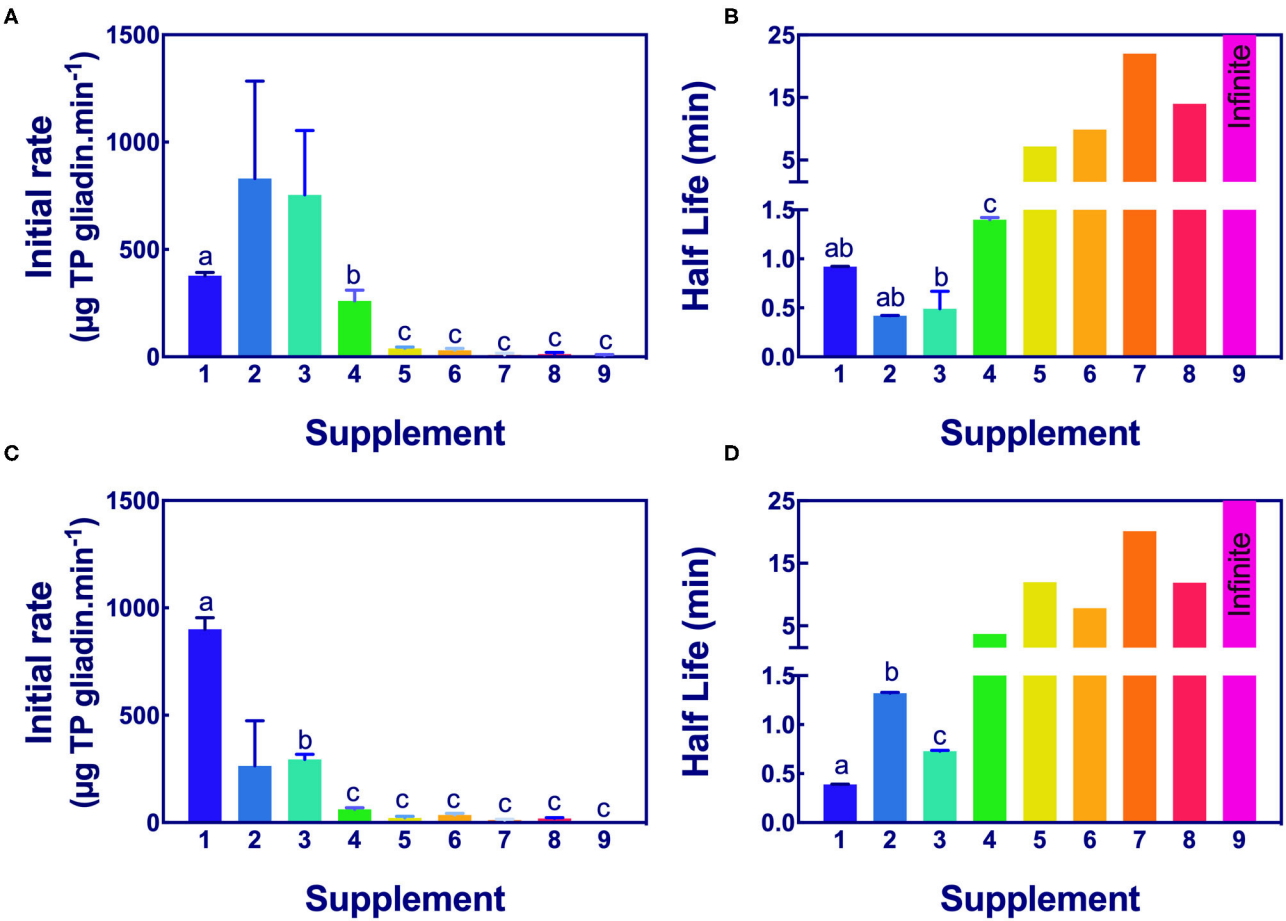
Significance of initial rate and half-life of epitope digestion estimated from Gluten-Tec ELISA. The initial rates **(A,C)** and half-lives **(B,D)** were calculated from analysis of Gluten-Tec ELISA data fitted to 1-phase decay, or where the data were noisy, as linear regression by Graph PAD Prism 8.0, at pH 3.5 **(A,B)** and pH 7.0 **(C,D)**. Rate (for simplicity gliadin consumption is shown here as positive), and half-life were normalized to an enzyme concentration of 1× . Mean (±SE) for constants are shown except where the half-life was calculated by linear regression. Half-lives shown as 25 min had infinite half-life. Where columns have different letters, the means were significantly different by one way ANOVA with Tukey's multiple comparison test (A, omitting columns 2 and 3 from the analysis, columns with different letters are significantly different at *p* < 0.05; B, different letters are significantly different at *p* < 0.007; C, omitting column 2, different letters are significantly different *p* < 0.005; D, consider only columns 1–3, *p* < 0.0001). ELISA, the enzyme-linked immunosorbent assay.

**Figure 6:**
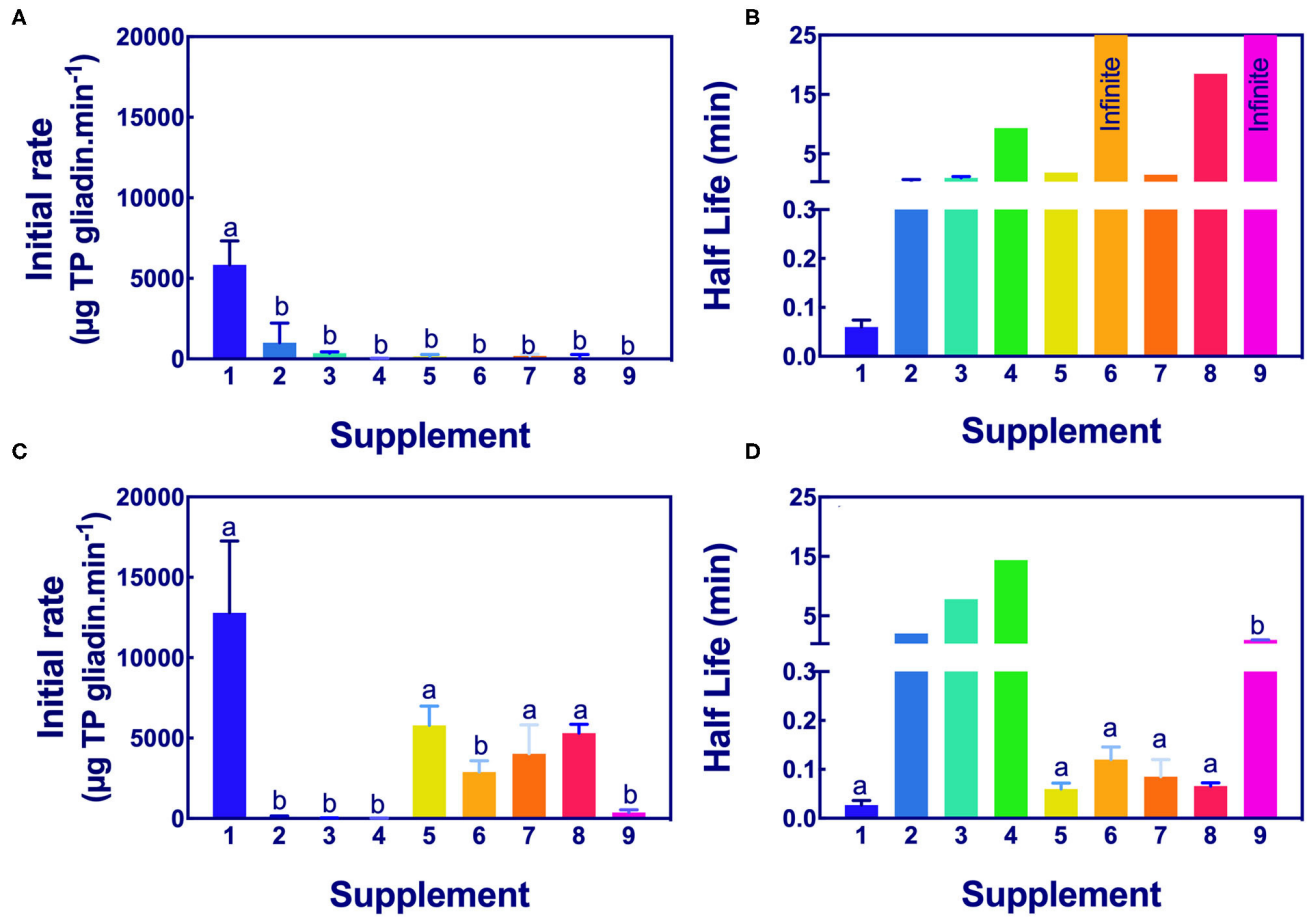
Significance of initial rate and half-life of epitope digestion estimated by Ridascreen ELISA analysis. The initial rates **(A,C)** and half-lives **(B,D)** were calculated from analysis of Ridascreen ELISA data fitted to 1-phase decay, or where the data were noisy as linear regression by Graph PAD Prism 8.0, at pH 3.5 **(A,B)** and pH 7.0 **(C,D)**. Rate (for simplicity gliadin consumption is shown here as positive), and half-life were normalized to an enzyme concentration of 1×. Mean (±SE) for constants are shown except where the half-life was calculated by linear regression. Reactions with infinite half-lives are shown as 25 min. Where columns have different letters, the means were significantly different by one-way ANOVA with Tukey's multiple comparison test (A, *p* < 0.009, LSD = 1834; B, no columns were significantly different; C, *p* < 0.04, LSD = 4760; D, omit columns 2, 3, and 4, *p* < 0.0001, LSD = 0.068). ELISA, the enzyme-linked immunosorbent assay.

The significance of the difference in the initial rates and half-lives of gliadin digestion were determined by ANOVA at pH 3.5 and pH 7.0 for Gluten-Tec ELISA (Figure 5) and Ridascreen ELISA (Figure 6).

*Aspergillus niger* prolyl endopeptidase containing enzymes 2 and 3 had the highest initial rate and shortest half-lives, followed by the caricain supplement 1, at about half the rate of supplement 2 and 3 but only when measured by Gluten-Tec ELISA at pH 3.5 (Figures 5A,B). The scatter of the initial rates of supplements 2 and 3 reduced the significance of the differences in mean values. The caricain containing supplement 1 had a significantly higher initial rate and shorter half-life (Figures 5C, 6C) than all other supplements when measured with Gluten-Tec at pH 7.0 (Figures 5C,D). The DPP-IV supplements 5, 6, 7, and 8 and the fungal/bacterial containing supplement 9 had little activity at either pH with Gluten-Tec ELISA.

The initial rates of the caricain supplement 1 were statistically larger, and the half-life was smaller than all other supplements when measured by Ridascreen ELISA at both pHs (Figure 6) excepting those of the DPP-IV supplements 5, 6, 7, and 8 which were not significantly different to caricain at pH 7.0 (Figures 6C,D). The fungal/ bacterial containing supplement 9 had little activity at either pH with Ridascreen ELISA.

## Discussion

There were significant differences in the rate of removal of celiac-relevant epitopes by commercially available enzyme supplements, *in vitro*.

The caricain supplement 1 most rapidly cleaved epitopes measured by Gluten-Tec ELISA at pH 7.0 and Ridascreen ELISAs at pH 3.5 and pH 7.0. Supplements 2 and 3 (AnPEP), which are released and active in the stomach, demonstrated strong activity at pH 3.5 against the epitopes measured by the Gluten-Tec ELISA, but showed minimal activity against the epitopes measured by Ridascreen ELISA. Supplements 4–8, all of which are released in the stomach, demonstrated minimal or no activity at pH 3.5 as measured by either ELISA, but had moderate activity at pH 7.0 when measured by Ridascreen ELISA. The fungal/bacterial containing supplement 9 had little activity under any conditions.

These results should be interpreted in light of the region of the digestive tract in which they are intended to work. Comparing the activity of supplements at their optimum pH, i.e., caricain and DPP-IV supplements all at pH 7, with AnPEP supplements at pH 3.5, all measured with Ridascreen ELISA may be used to judge the relative rates of cleavage (Table 2). The reasons for concentrating on the Ridascreen assay are 2-fold: Ridascreen ELISA is the only Codex-approved method for measuring gluten. Secondly, we suggest Ridascreen ELISA is a better proxy for total gluten consumption. Ridascreen antibodies detect several coeliac relevant epitopes, QQPFP, QQQFP, LQPFP, and QLPFP. These epitopes occur at multiple locations on each gluten protein, and they occur on many different gluten proteins (Supplementary Figure 5). In contrast, the Gluten-Tec ELISA is a more specific reagent and detects the single T-cell stimulatory epitope of Glia-α20, PFRPQQPYPQ (4). This epitope is only present in one location on each gliadin protein but is not present in all gliadin proteins (Supplementary Figure 5). The Gluten-Tec ELISA was included in the study to provide additional data on the fate of a clinically important epitope. Both Gluten-Tec and Ridascreen ELISA epitopes are disabled by internal cleavage of any of the P and Q amino acids in the epitope.

**Table 2:** Relative kinetics of digestion of coeliac epitopes at optimum pH measured with Ridascreen ELISA.

**Supplement (pH)**	**1 (pH 7.0)**	**2 (pH 3.5)**	**3 (pH 3.5)**	**5 (pH 7.0)**	**6 (pH 7.0)**	**7 (pH 7.0)**	**8 (pH 7.0)**
Initial Rate ± SE^1^ (μg TP gliadin/min)	−12,800^a^ ± 4,500	−1,000^b^ ± 1,200	−350^b^ ± 90	−5,800^a^ ± 1,200	−2,900^c^ ± 700	−4,000^a^ ± 1,800	−5,300^a^ ± 560
Half life ± SE^1^ (min)	0.027^a^ ± 0.009	0.33^a^ ± 0.37	0.97^b^ ± 0.22	0.060^a^ ± 0.012	0.12^a^ ± 0.026	0.085^a^ ± 0.035	0.066^a^ ± 0.006

1 *Initial rate and half-life were corrected to 1× enzyme concentration. A negative rate correlates to gliadin consumption. Initial rates with different letters are significantly different by one-way ANOVA with Tukey's multiple comparison test (p <0.08). When half-lives were compared, those for supplements 1, 5, 6, 7, 8 at pH 7.0, differed significantly from the half-life of supplements 3 (p < 0.07) at pH 3.5. ELISA, the enzyme-linked immunosorbent assay*.

For this comparison, the initial rate for supplement 1, measured by Ridascreen ELISA at pH 7, was significantly faster (*p* < 0.04) than that for supplements 2 and 3 measured at pH 3.5 with Ridascreen ELISA (Table 2). The initial rates for DPP-IV supplements 5 and 7, and the fungal/bacterial proteases in supplement 8 at pH 7.0, showed moderate initial rates, which did not differ significantly from that of supplement 1 (*p* > 0.2). However, the initial rate of DPP-IV supplement 6 at pH 7.0 differed significantly from those of all other supplements. The half-life of supplement 1 at pH 7.0 was lower than for supplement 3 measured at pH 3.5 with Ridascreen ELISA (*p* > 0.05), the remaining half-lives did not differ significantly. The final supplement (9) did not show any significant rate of epitope removal and was not discussed.

Supplement 1 was the only supplement to employ an enterically coated tablet designed to dissolve and release the enzyme only in the neutral pH of the duodenum and small intestine (51). The results obtained for supplement 1 at pH 3.5 are therefore largely irrelevant to its performance *in vivo*. The developers of this supplement chose to use an enteric coating to target the release of caricain to the small intestine, the site where gluten peptides initiate symptoms in coeliacs. We demonstrate here that caricain is stable to pepsin at pH 3 and trypsin at pH 7 (Supplementary Figure 4). However, at pH 2, caricain undergoes a conformational transition leading to instability and rapid degradation by pepsin (52). As the pH in the stomach can fall as low as pH 1.0 immediately after consuming food (53), it is apparent that the enteric coating is required for a supplement containing caricain.

Supplements other than caricain that were investigated were provided in capsules that break down and release their enzymes in the stomach. The primary site of AnPEP action is the stomach, an acidic environment in the presence of pepsin. *Aspergillus oryzae* DPP-IV also released into the stomach is stable to acidic pepsin but inactive at stomach pH (29, 31). It is therefore likely that DPP-IV enzymes released in the stomach pass into the pH neutral environment of the duodenum/small intestine. Unless exhausted or inhibited in the stomach, it is expected that they should remain optimally active in this secondary site.

For an enzyme supplement to be useful in alleviating symptoms induced by celiac immuno-toxic gluten peptides, all ingested peptides should be completely cleaved. In addition to ELISA, this requires investigation with HPLC, MS, and T cells to document the concentration and toxicity of coeliac relevant epitopes remaining after digestion. HPLC-MS/MS analysis of gliadin digests following the action of supplements is underway and will be reported elsewhere.

## Conclusion

Of the nine dietary protease supplements tested, supplement 1, at pH 7.0, most rapidly digested the key immuno-reactive gluten epitopes associated with the pathology of CD in PT-gliadin. This was identified by the R5 antibody in the Codex-approved competitive Ridascreen ELISA as well as the Gluten-Tec ELISA. None of the other eight supplements demonstrated the ability to digest the combined epitopes measured by the two ELISA.

Results from prior clinical studies suggest that the caricain containing supplement 1 (GluteGuard) may be a useful adjunct to a gluten-free diet as a means of protecting those with CD and other gluten sensitivities from inadvertent dietary gluten contamination.

## Data Availability Statement

The original contributions presented in the study are included in the article/Supplementary Material, further inquiries can be directed to the corresponding author.

## Author Contributions

GT conceived and carried out the experiments, analyzed the data, and wrote the manuscript.

## Funding

Funding for this research was provided by Glutagen Pty Ltd. which had no role in study design, data collection, and analysis, or the decision to publish. A preliminary version of this data was presented at the Dietitians Association of Australia, Gold Coast, Aug 2019 and the International Celiac Disease Symposium, Sept 2019, Paris.

## Conflict of Interest

The author declares that the research was conducted in the absence of any commercial or financial relationships that could be construed as a potential conflict of interest.

## Publisher's Note

All claims expressed in this article are solely those of the authors and do not necessarily represent those of their affiliated organizations, or those of the publisher, the editors and the reviewers. Any product that may be evaluated in this article, or claim that may be made by its manufacturer, is not guaranteed or endorsed by the publisher.
