# Supplementary material for: Relative Rates of Gluten Digestion by Nine Commercial Dietary Digestive Supplements
Source: Front Nutr. 2021 Dec 7;8:784850. doi: 10.3389/fnut.2021.784850 (PMC8688929; doi:10.3389/fnut.2021.784850)
Supplement: Supplementary file 1 [file Data_Sheet_1.docx]

Supplementary Material

## Supplementary Tables

**Supplementary Table 1: Rates and half-life of epitope digestion at pH 3.5, estimated by GlutenTec ELISA**

| **Supplement** | **1** | **2** | **3** | **4** | **5** | **6** | **7** | **8** | **9** |
| --- | --- | --- | --- | --- | --- | --- | --- | --- | --- |
| **Initial Rate ± SE^1^ (µg TP gliadin/min)** | -380  ±15 | -830  ±450 | -750  ±300 | -260  ±50 | -40  ±6 | -30  ±9 | -10  ±8 | -14  ±7 | 6  ±4 |
| **Half Life1 ± SE^1,2^**  **(min)** | 0.92  ±0.003 | 0.42  ±0.002 | 0.49  ±0.18 | 1.4  ±0.02 | 7.2^2^ | 9.9^2^ | 220^2^ | 140^2^ | NA^3^ |
| **r^2^** | 0.998 | 0.997 | 0.977 | 0.967 | 0.805 | 0.689 | 0.19 | 0.30 | 0.19 |
| **Protein concentration^1^ (mg/mL)** | 1.22  ±0.1 | 0.51  ±0.04 | 0.59  ±0.02 | 0.23  ±0.01 | 0.71  ±0.03 | 1.19  ±0.03 | 1.28  ±0.01 | 0.85  ±0.03 | 1.29  ±0.08 |

1: Corrected to 1x enzyme concentration. Negative rate correlates to gliadin consumption.

2: Initial rate and half-life for reactions 1, 2, 3, 4 were calculated by non-linear regression (1 phase decay) and reactions 5 - 9 were too variable to meaningfully analyze by non-linear regression and were analyzed by linear regression – this did not allow estimation of the SE of half-life. The r2 value of the regression is shown.

3: NA Rate is slightly positive, i.e. half-life is infinite.

**Supplementary Table 2: Rates and half-life of epitope digestion at pH 7.0, estimated by GlutenTec ELISA**

| **Supplement** | **1** | **2** | **3** | **4** | **5** | **6** | **7** | **8** | **9** |
| --- | --- | --- | --- | --- | --- | --- | --- | --- | --- |
| **Initial Rate ± SE^1^**  **(µg TP gliadin/min)** | -900  ±60 | -260  ±210 | -290  ±20 | -61  ±8 | -22  ±8 | -36  ±7 | -11  ±4 | -20  ±4 | 0.10  ±5 |
| **Half Life1 ± SE^1,2^**  **(min)** | 0.39±  0.002 | 1.32±  0.009 | 0.73±  0.01 | 3.7^2^ | 12^2^ | 7.8^2^ | 20^2^ | 12^2^ | NA^3^ |
| **r^2^** | 0.996 | 0.994 | 0.92 | 0.84 | 0.48 | 0.79 | 0.46 | 0.80 | <0.1 |

1: Corrected to 1x enzyme concentration. Negative rate correlates to gliadin consumption.

2: Initial rate and half-life for reactions 1, 2, 3 were calculated by non-linear regression (1 phase decay) and reactions 4, 5, 6, 7, 8, 9 were too variable to meaningfully analyze by non-linear regression and were analyzed by linear regression – this did not allow estimation of the SE of half-life. The r2 value of the regression is shown.

3: NA, Rate is slightly positive, i.e. half-life is infinite.

**Supplementary Table 3. Rates and half-life of epitope digestion at pH 3.5, estimated by Ridascreen ELISA**

| **Supplement** | **1** | **2** | **3** | **4** | **5** | **6** | **7** | **8** | **9** |
| --- | --- | --- | --- | --- | --- | --- | --- | --- | --- |
| **Initial Rate ± SE^1^ (µg TP gliadin/min)** | -5,850  ±1,500 | -1,010  ±1,200 | -350  ±90 | -31  ±9 | -151  ±130 | 0.00 | 200  ±100 | -15  ±140 | 0.00 |
| **Half Life* ± SE^1,2^**  **(min)** | 0.06  ±0.01 | 0.3  ±0.4 | 1.0  ±0.2 | 9.3^2^ | 1.9^2^ | Infinite^3^ | 1.5^2^ | 19^2^ | Infinite^3^ |
| **r2** | 0.95 | 0.81 | 0.96 | 0.72 | 0.19 | 1.0 | 0.68 | 0.0006 | 1.000 |

1: Corrected to 1x enzyme concentration. Negative rate correlates to gliadin consumption.

2: Initial rate and half-life for reactions 1, 2, 3 were calculated by non-linear regression (1 phase decay) and reactions 4- 9 were too variable to meaningfully analyze by non-linear regression and were calculated by linear regression – this did not allow estimation of the SE of half-life. The r2 value of the regression is shown.

3: NA, Rate is zero, i.e. half-life is infinite.

**Supplementary Table 4. Rates and half-life of epitope digestion at pH 7.0 estimated by Ridascreen ELISA.**

| **Supplement** | **1** | **2** | **3** | **4** | **5** | **6** | **7** | **8** | **9** |
| --- | --- | --- | --- | --- | --- | --- | --- | --- | --- |
| **Initial Rate ± SE^1^**  **(µg TP gliadin/min)** | -12,800  ±4,500 | -120  ±50 | -30  ±6 | -16  ±16 | -5,800  ±1,200 | -2,900  ±700 | -4,000  ±1,800 | -5,300  ±560 | -380  ±160 |
| **Half Life* ± SE^1,2^**  **(min)** | 0.027  ±0.009 | 2.03^2^ | 7.8^2^ | 14.4^2^ | 0.060  ± 0.01 | 0.12  ±0.03 | 0.09  ±0.04 | 0.066  ±0.006 | 0.94  ±0.04 |
| **r^2^** | 0.943 | 0.54 | 0.86 | 0.23 | 0.98 | 0.97 | 0.88 | 0.99 | 0.89 |

1: Corrected to 1x enzyme concentration. Negative rate correlates to gliadin consumption.

2: Initial rate and half-life for reactions 1, 5, 6, 7, 8, 9 were calculated by non-linear regression (1 phase decay) and reactions 2, 3, and 4 were too variable to meaningfully analyze by non-linear regression and were analyzed by linear regression – this did not allow estimation of the SE of half-life. The r2 value of the regression is shown.

## Supplementary Figures

**
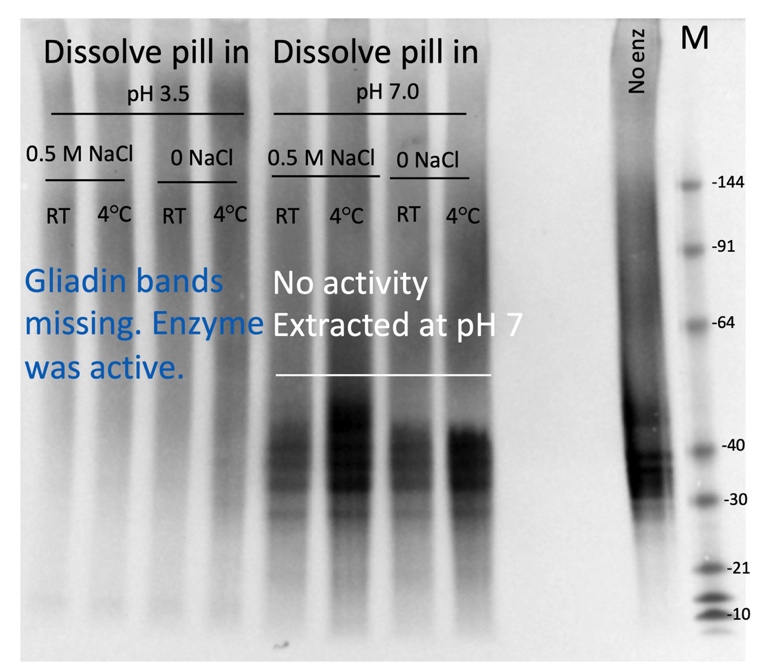
**

**Supplementary Figure S1. Activity of supplement 2 (AnPEP) was detected only when extracted into pH 3 buffer.**

Supplement 2 (ANPEP enzyme) was dissolved at either pH 3.5 or pH 7.0, plus a final concentration of 0 or 0.5 mole/L NaCl, at RT or 4C and enzyme activity assayed at a final concentration of 1.1 x. At this high enzyme concentration native gliadin (500 µg) was digested (pH 3.5). Digestion was measured by western blot, developed with Sigma anti-gliadin HRP at 1/1,000x, and calibrated with prestained standards (M; Invitrogen). Digestion was only seen when the preparation was extracted at pH 3.5.


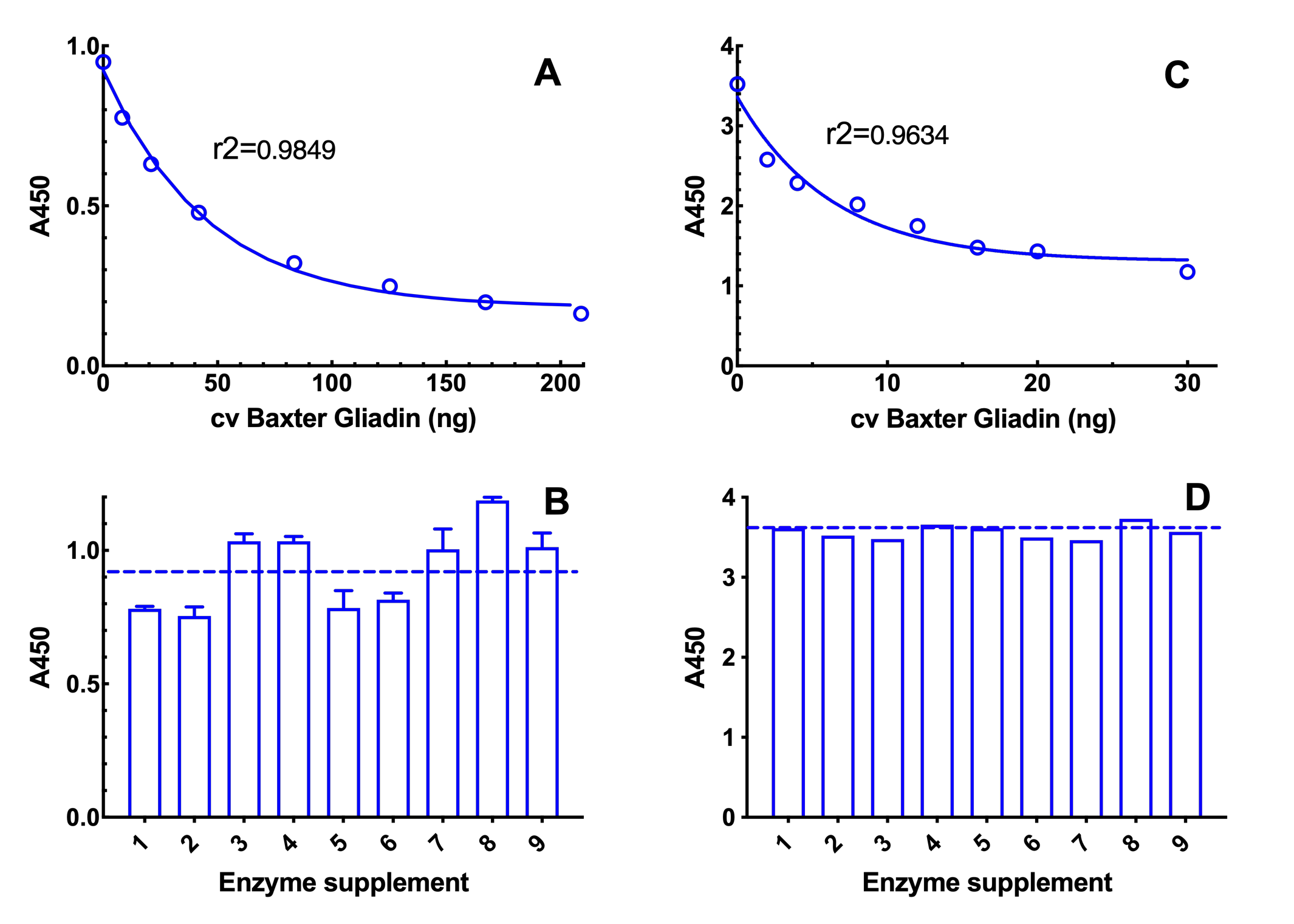


**Supplementary Figure S2.** **Controls for ELISA antibodies.**

(A) cv Baxter gliadin standard curve with Ridascreen ELISA (A), and (B) enzyme supplement alone showing slight damage to Ridascreen ELISA due to residual proteolytic activity after heat treatment of diluted enzyme assays for 10 min at 95C. The dotted lines show the zero-addition standard and is the maximum signal level expected if there was no damage to the ELISA system. (C) cv Baxter gliadin standard curve with Gluten-Tec ELISA, and (D) enzyme supplement alone showing slight damage to Gluten Tec-ELISA due to residual proteolytic activity after heat treatment of enzymes for 10 min at 95C. The dotted line shows the zero-addition standard and is the maximum signal level expected if there was no damage to the Gluten-Tec antibodies. There was no damage of either ELISA system after heating dilute enzyme aliquots at 20 min at 95C.


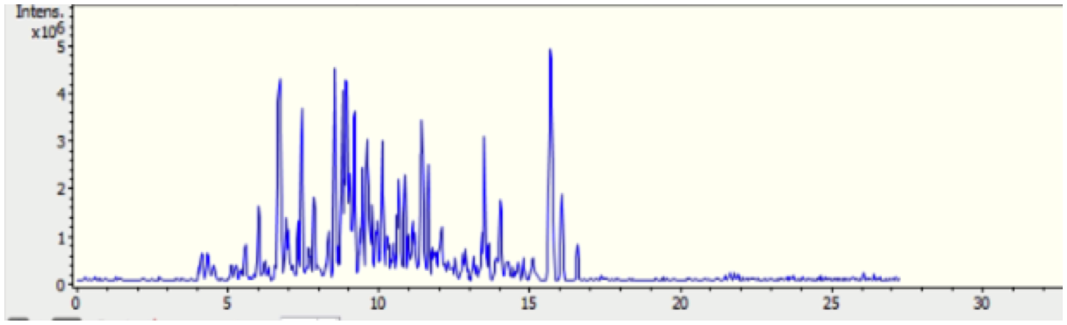


**Supplementary Figure S3. HPLC of TP-digested Baxter gliadin.**

HPLC analysis of TP-gliadin showed over 30 peptides, confirming adequate hydrolysis of the native gliadin. A Shimadzu (150 mm x 3.0 mm I.D., 3 um, C8) column was eluted at 0.5 ml/min with a linear gradient from solvent A (0.1% (v/v) trifluoroacetic acid (TFA, 5% (v/v) aqueous acetonitrile) to solvent B (0.1% TFA in 60% (v/v) aqueous acetonitrile). This showed that the digestion had produced many small peptides.

***
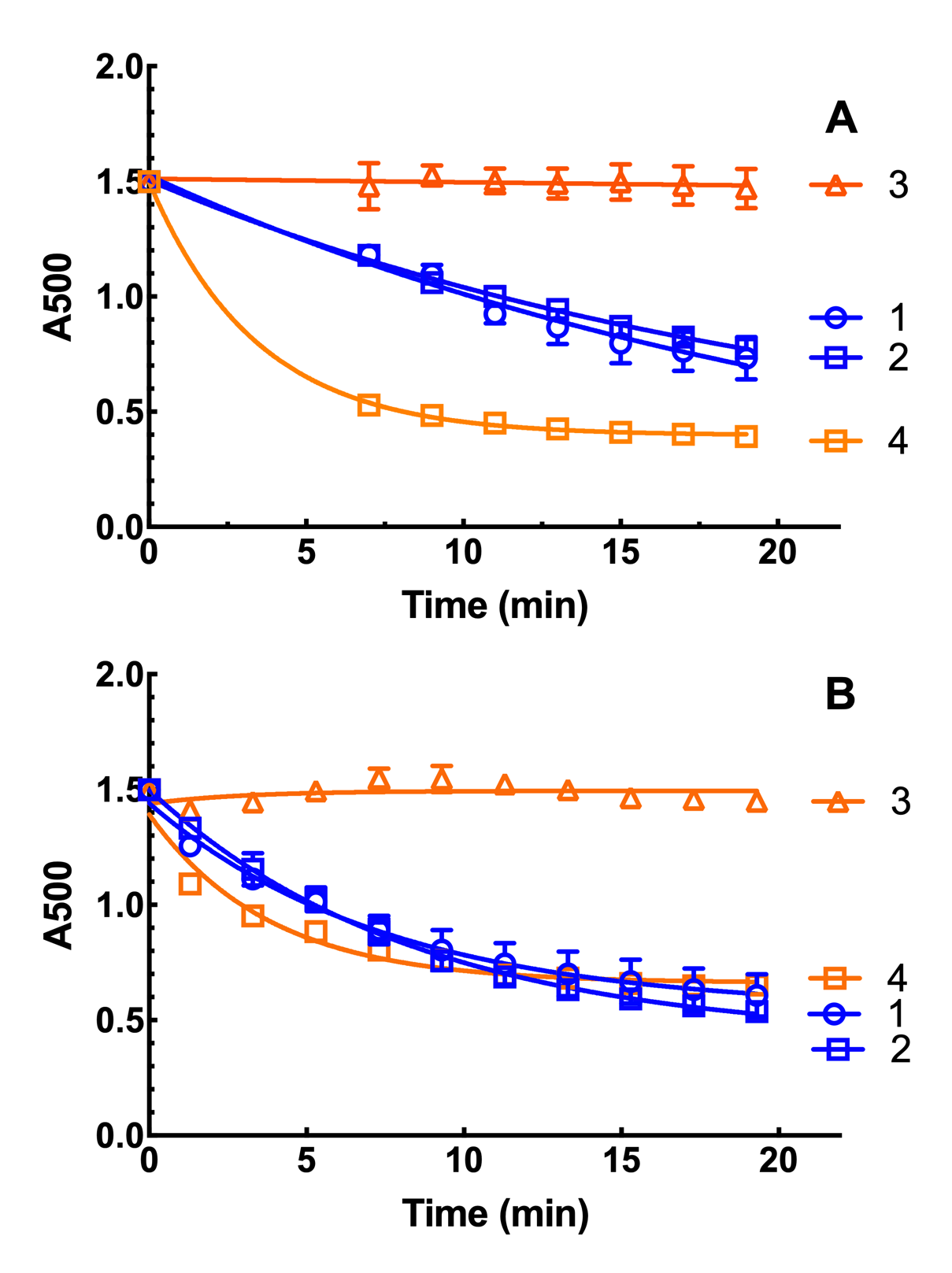
***

**Supplementary Figure S4.** **Supplement 1 was stable to pepsin and trypsin activity.**

Supplement 1 was dissolved and added to trypsin (A) at pH 7, at a final enzyme concentration of 0.011x and incubated for 10 min then trypsin inhibitor added, and the reaction diluted by the addition of MM7 and gliadin digestion started by the addition of native gliadin (Sigma) (A1). Inactive trypsin controls had trypsin inhibitor added before the enzyme (A2), or without the enzyme (A3). Active trypsin control had no supplement 1, and no trypsin inhibitor (A4). Alternately supplement 1 was added to pepsin (B) at pH 3.5 and incubated as above before the pH was adjusted to pH 7 and gliadin added as above (B1). Inactive pepsin controls had the pH adjusted to pH 7 before the addition of supplement 1 (B2) or no enzyme supplement (B3). Active pepsin control remained at pH 3.5 (B4). When gliadin was added to the solution it formed a precipitate increasing the absorbance. As proteolysis proceeded the insoluble precipitate was hydrolysed into shorter soluble peptides causing a decrease in light scattering at A550. Digestion of gliadin was followed by the decrease in light scattering at A550. Mean (±SE) is shown, except when error was smaller than symbol size. Duplicate points are modelled by either linear regression (for straight lines) or by non-linear regression (for curves). The ratio of trypsin to supplement 1 was 1:1 on a protein basis – this is well in excess of the amount of trypsin/ pepsin normally used. Normally trypsin digests for example are carried out at a ratio of 1: 20 protease: target protein. Similar results were seen when the proteases were increased dramatically to a ratio of 100:1 protease: supplement1.


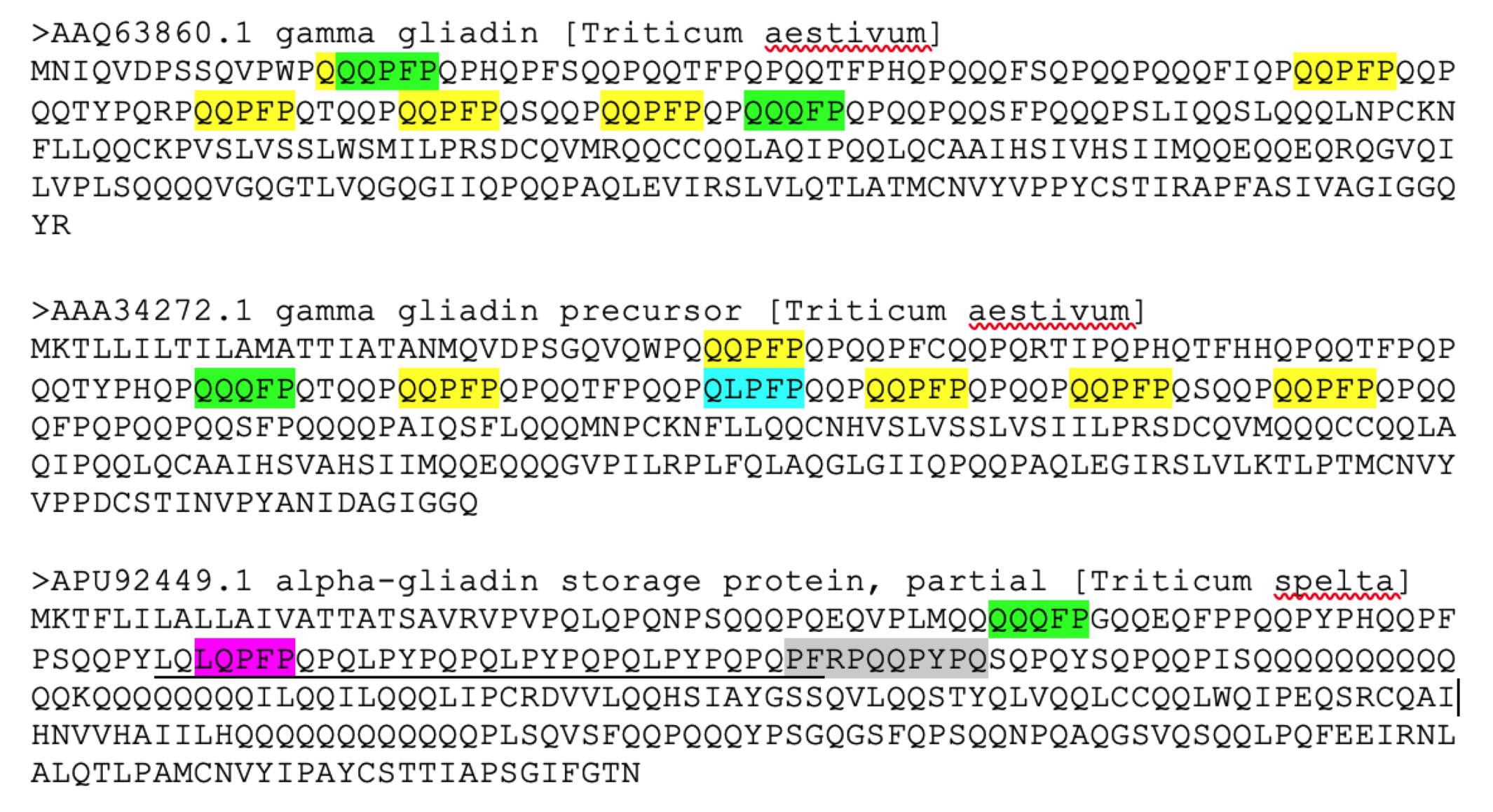


**Supplementary Figure S5. Frequency of epitopes in three representative gliadin proteins.** The Gluten Tec epitope, Glia-α20, is shown as PFRPQQPYPQ; Ridascreen epitopes: QQPFP, QQQFP, LQPFP and QLPFP; and the α2-gliadin^56-58^ 33 mer: LQLQPFPQPQLPYPQPQLPYPQPQLPYPQPQPF
